# Accuracy of cone-beam computed tomography, digital mammography and digital breast tomosynthesis for microcalcifications and margins to microcalcifications in breast specimens

**DOI:** 10.1038/s41598-022-21616-3

**Published:** 2022-10-21

**Authors:** Claudia Neubauer, Jannina Samantha Yilmaz, Peter Bronsert, Martin Pichotka, Fabian Bamberg, Marisa Windfuhr-Blum, Thalia Erbes, Jakob Neubauer

**Affiliations:** 1 Department of Radiology, Medical Center, University of Freiburg, Faculty of Medicine, University of Freiburg, Freiburg im Breisgau, Germany; 2 Institute for Surgical Pathology, Medical Center, University of Freiburg, Faculty of Medicine, University of Freiburg, Freiburg im Breisgau, Germany; 3 Tumorbank Comprehensive Cancer Center Freiburg, Medical Center, University of Freiburg, Faculty of Medicine, University of Freiburg, Freiburg Im Breisgau, Germany; 4 Core Facility for Histopathology and Digital Pathology, Medical Center, University of Freiburg, Faculty of Medicine, University of Freiburg, Freiburg Im Breisgau, Germany; 5 Medical Physics, Department of Radiology, Medical Center, University of Freiburg, Faculty of Medicine, University of Freiburg, Freiburg im Breisgau, Germany; 6 Department of Obstetrics and Gynecology, Medical Center, University of Freiburg, Faculty of Medicine, University of Freiburg, Freiburg im Breisgau, Germany

**Keywords:** Breast cancer, Cancer imaging

## Abstract

Accurate determination of resection margins in breast specimens is important as complete removal of malignancy is a prerequisite for patients’ outcome. Mammography (DM) as 2D-technique provides only limited value in margin assessment. Therefore, we investigated whether cone-beam computed tomography (CBCT) or digital breast tomosynthesis (DBT) has incremental value in assessing margins to microcalcifications. Three independent readers investigated breast specimens for presence of microcalcifications and the smallest distance to margins. Histopathology served as gold standard. Microcalcifications were detected in 15 out of 21 included specimens (71%). Pooled sensitivity for DM, DBT and CBCT for microcalcifications compared to preoperative DM was 0.98 (CI 0.94–0.99), 0.83 (CI 0.73–0.94) and 0.94 (CI 0.87–0.99), pooled specificity was 0.99 (CI 0.99–0.99), 0.73 (CI 0.51–0.96) and 0.60 (CI 0.35–0.85). Mean measurement error for margin determination for DM, DBT and CBCT was 10 mm, 14 mm and 6 mm (*p* = 0.002) with significant difference between CBCT and the other devices (*p* < 0.03). Mean reading time required by the readers to analyze DM, DBT and CBCT, was 36, 43 and 54 s (*p* < 0.001). Although DM allows reliable detection of microcalcifications, measurement of resection margin was significantly more accurate with CBCT. Thus, a combination of methods or improved CBCT might provide a more accurate determination of disease-free margins in breast specimens.

## Introduction

Microcalcifications in the breast can be an indicator of malignancy^1–3^. In about 32% of cases, microcalcifications are the only imaging feature of ductal carcinoma in situ (DCIS) or invasive cancer of the breast^4^. Currently, the most important standard method for detecting breast microcalcifications is mammography, which is recommended as a cancer screening measure^5^ and for women with a suspected breast tumor. Suspicious microcalcifications in the breast can be evaluated by vacuum biopsies with histopathological examination^6^. If malignancy is histologically confirmed, additional surgery is performed. The completeness of removal of all suspicious microcalcifications can be controlled by specimen mammography during surgery.

However, mammography is a two-dimensional examination technique, which is only suitable to a limited extent for tumor size measurement^7^ and the examination of mostly amorphous resected tissue especially in dense breast tissue^8^. Due to superimposition phenomena, it is only possible to make limited statements about the tumor extension or the distances between microcalcifications and resection margin. Nevertheless, the complete removal of tumor tissue is one of the most important predictors for patients’ outcome^9,10^. From these points of view, it seems very reasonable to utilize three-dimensional examination techniques for the evaluation of resected specimens.

Therefore, the presented study applies the three-dimensional imaging methods digital breast tomosynthesis (DBT) and cone-beam computed tomography (CBCT) in addition to digital mammography (DM). DBT represents a mammographic technique, involving mammograms from different angles to create computed tomosynthesis layered reconstructions of the breast^11,12^. Especially in the case of inhomogeneously dense breast parenchyma, DBT shows diagnostic advantages over conventional DM^8,13–17^. In detection of microcalcifications it shows similar results as DM^18–22^. However, due to a limited scan angle DBT cannot produce isotropic imaging data. In contrast, CBCT is a fully three-dimensional imaging technique without breast compression, which allows reconstructions with isotropic voxels and a high spatial resolution that even enables the visualization of microcalcifications to different extents, unlike conventional computed tomography^23–25^. In addition, in other studies dedicated breast CBCT dose proved to be similar to or even less than DM dose^26^.

In this study, we aimed to investigate whether CBCT has additional value over DM and DBT in assessing resection margins of breast specimens with microcalcifications. Our hypothesis was that the CBCT had a lower error in determining the resection margin compared to the other two modalities.

## Results

### Cohort characteristics

**Figure 1:**
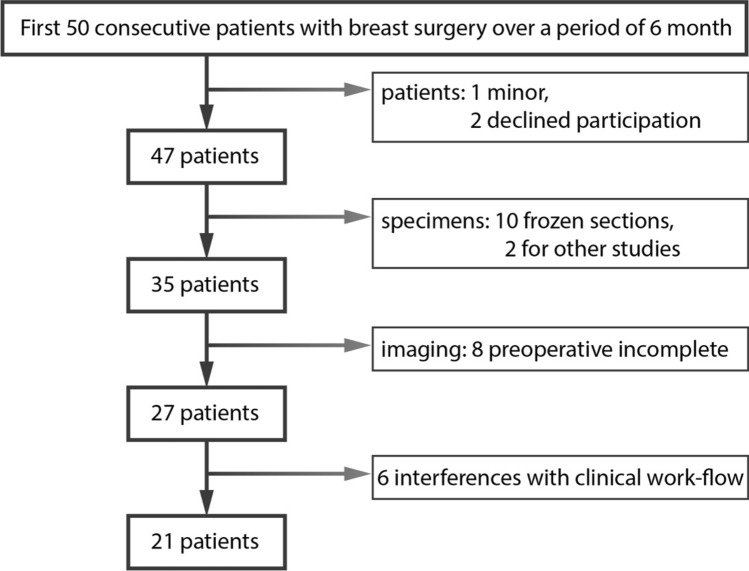
Eligible patients and exclusion criteria resulting in the participation of 21 patients.

For our study we selected the first 50 patients who received breast surgery in our institution over a period of 6 months. After applying all exclusion criteria 21 patients could finally be included in the presented study (more details for patient inclusion are given in Fig. 1). Patients were between 24 and 69 years old. Specimens were withdrawn from the right (*n* = 7) and left (*n* = 14) breast.

### Histopathological findings

Patients’ pre-operative proven histopathologic diagnoses included DCIS, invasive carcinoma, a combination of both and fibrocystic lesions and are provided in Table 1. Microcalcifications were detected in 15 resected specimens according to mammography as the reference standard.

**Table 1:** Frequency of pathologic diagnoses in mammography in our study collective (*DCIS* ductal carcinoma in situ, *MC* microcalcifications, *wo* without).

Pathology	Number of patients with MC	Number of patients wo MC
Invasive Carcinoma	2 (9,5%)	2 (9,5%)
Invasive Carcinoma with DCIS	3 (14,3%)	0
DCIS	8 (38%)	0
Fibrocystic lesion	2 (9,5%)	4 (19%)

### Measures of diagnostic performance

The pooled sensitivity for DM, DBT and CBCT for detection of microcalcifications in specimens in comparison to the preoperative DM (Table 2) were 0.98 (CI 0.94–0.99), 0.83 (CI 0.73–0.94) and 0.94 (CI 0.87–0.99). The pooled specificity for DM, DBT and CBCT for microcalcifications (Table 2) were 0.99 (CI 0.99–0.99), 0.73 (CI 0.51–0.96) and 0.60 (CI 0.35–0.85).

**Table 2:** The pooled sensitivity, specificity and mean measurement errors for the smallest distance of delineated microcalcifications to the resection margin for digital mammography (DM), digital breast tomosynthesis (DBT) and cone-beam computed tomography (CBCT) (*CI* confidence interval, *SD* standard deviation).

	Sensitivity	Specificity	Mean error for distance to margin
DM	0.98 (CI 0.94–0.99)	0.99 (CI 0.99–0.99)	10 mm (SD 14 mm)
DBT	0.83 (CI 0.73–0.94)	0.73 (CI 0.51–0.96)	14 mm (SD 20 mm)
CBCT	0.94 (CI 0.87–0.99)	0.60 (CI 0.35–0.85)	6 mm (SD 8 mm)

The mean maximal size of microcalcifications measured in the DM of the specimens was 0.37 mm (SD 0.08). Two specimens showed single clusters of microcalcifications that measured up to 0.79 and 0.86 mm.

**Figure 2:**
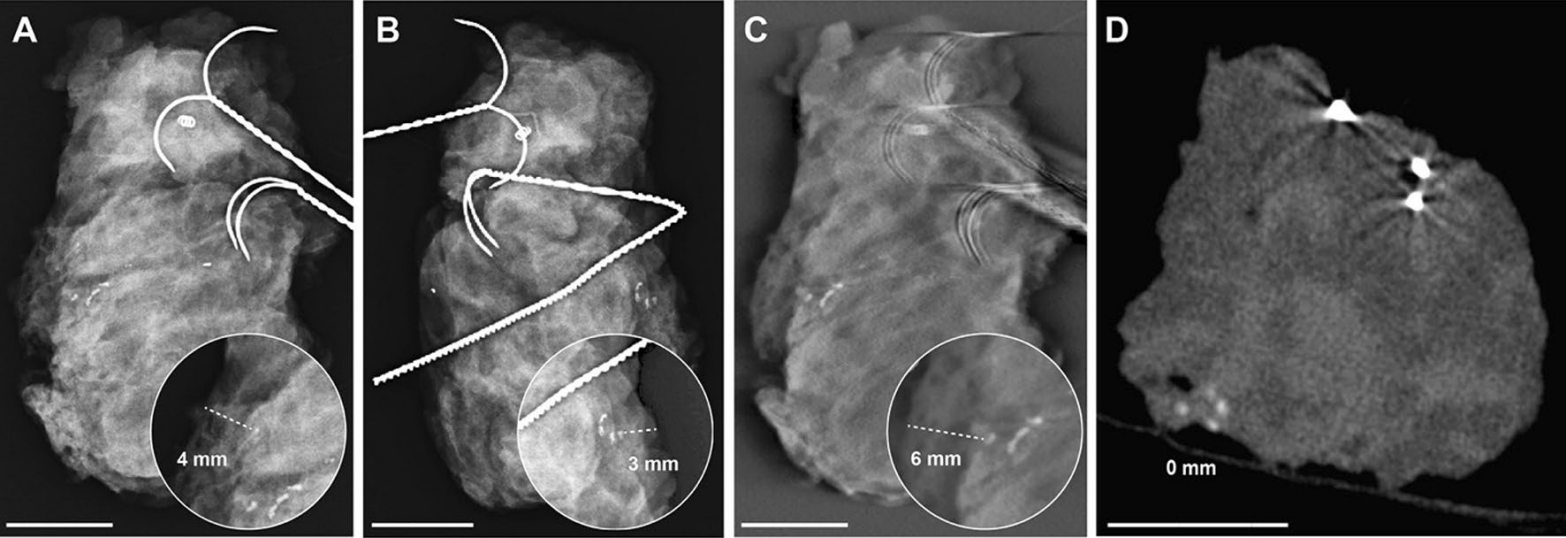
Breast specimen with microcalcifications, histopathologically proven as invasive carcinoma of no special type, clip and wires in (**A**) and (**B**) the two standard projections of specimen mammography (DM), (**C**) in digital breast tomosynthesis (DBT) and (**D**) cone-beam computed tomography (CBCT) imaging. Whilst the microcalcifications project 4 or 3 mm from the margin in DM (dependent of projection), there are 6 mm distance from the margin in DBT as the smaller calcification was not reliably detected in the blinded measurements and in the CBCT the microcalcifications could be clearly localized directly at the margin due to real 3D placement and reconstructions. Some clear metal wear [middle of (**A**) and close to middle right margin in (**B**)] should not be mistaken for microcalcification. In addition, metal-related artifacts around the wires are seen in (**C**) DBT and (**D**) CBCT images. The white scalebar indicates 1 cm.

**Figure 3:**
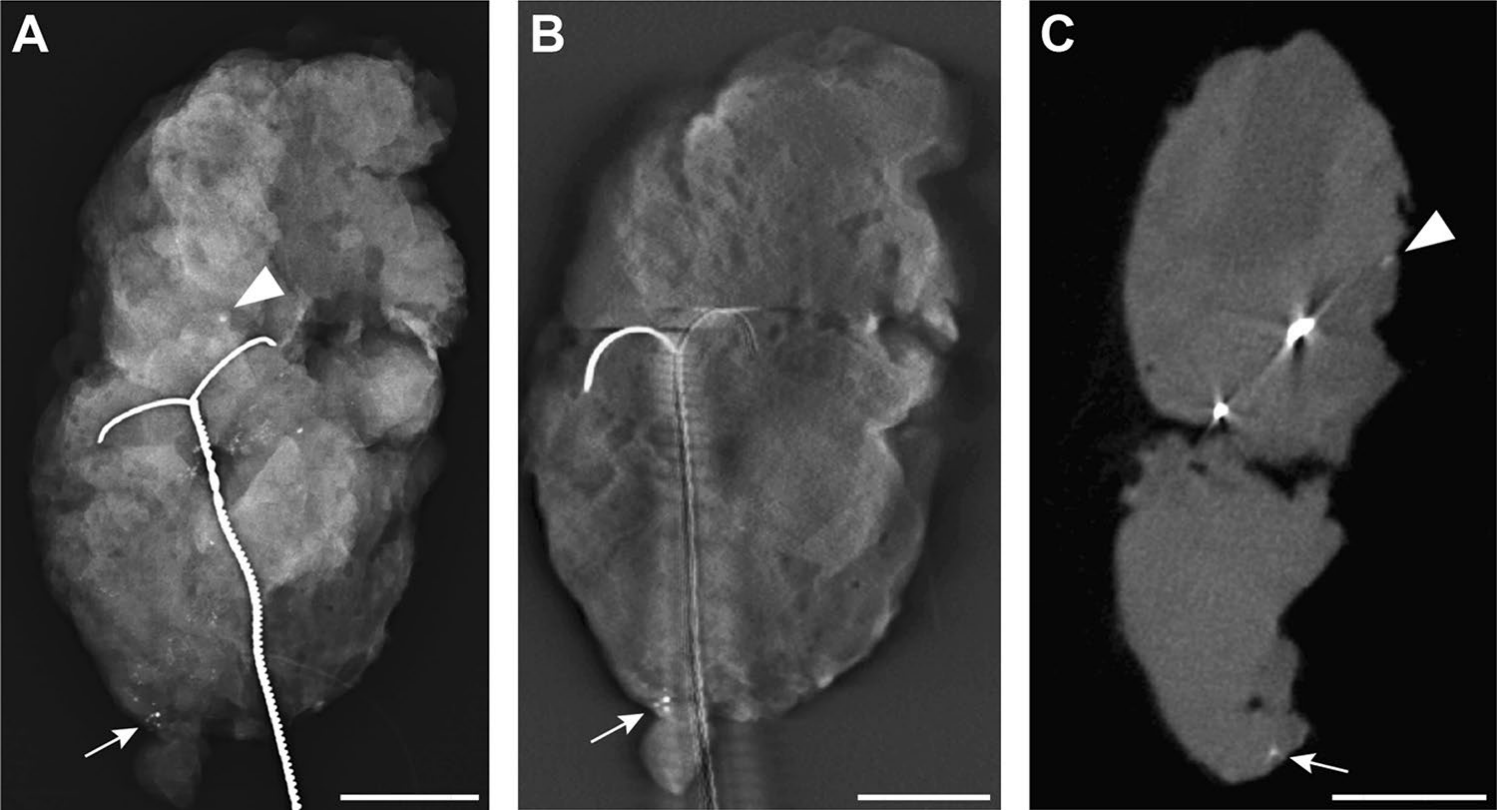
Breast specimen with microcalcifications, histopathologically proven as fibrocystic lesion with usual ductal hyperplasia, in (**A**) one projection of mammography (DM), (**B**) the same projection of digital breast tomosynthesis (DBT) and (**C**) cone-beam computed tomography (CBCT). Although microcalcifications can be detected directly at the lower margin in all modalities (arrow), there are other microcalcifications that are only clearly detected in DM and CBCT (arrowhead). Here, direct contact to the margin is exclusively seen in the CBCT (arrowhead). Again, metal-related artifacts around the wires are seen in (**B**) DBT and (**C**) CBCT images. The white scalebar indicates 1 cm.

The mean measurement errors for the smallest distance of delineated microcalcifications to the resection margin for DM, DBT and CBCT (Table 2) were 10 mm, 14 mm and 6 mm (*p* = 0.002). There was a significant difference between CBCT and the other devices (CBCT vs. DM with *p* = 0.03 and CBCT vs. DBT with *p* = 0.006). No significant difference was observed between DM and DBT (*p* = 0.81). Imaging examples of two representative specimens are provided in Figs. 2 and 3. Both show clear differences in the projection of microcalcifications in relation to the resection margins between the three modalities.

The mean absorbed dose for DM, DBT and CBCT was 109 µSv, 132 µSv and 2167 µSv (SD 5, 18, 112).

### Inter-rater reliability, reading time and confidence

The inter-rater reliability for resection margin measurements was 0.88 (CI 0.73–0.95), 0.93 (CI 0.82–0.98) and 0.78 (CI 0.57–0.92) for DM, DBT and CBCT.

The mean reading time for DM, DBT and CBCT was 36, 43 and 54 s. There was a significant difference in reading time between CBCT and DM (*p* < 0.001), also between CBCT and DBT (*p* = 0.03). However, no significant difference in reading time was detected between DM and DBT (*p* = 0.12).

The mean confidence in detection of microcalcifications (Likert scale 1–4 with 1 being very safe and 4 being very uncertain) was 1.1, 1.7 and 1.5 for DM, DBT and CBCT (*p* < 0.001). There was a significant difference between DM and DBT (*p* = 0.006), but no significant difference between the others.

The mean confidence for measurement of the smallest distance of delineated microcalcifications to the resection margin (Likert scale 1–4 with 1 being very safe and 4 being very uncertain) was 2.0, 2.0 and 1.8 for DM, DBT and CBCT (*p* = 0.15) without any significant differences between the modalities.

## Discussion

In our study, after breast surgery DM showed the highest sensitivity and specificity for the detection of microcalcifications in specimens in comparison to the reference standard of preoperative DM. One reason to explain this advantage of DM over DBT with the lowest sensitivity and over CBCT with the lowest specificity might be explained due to the better spatial resolution of DM especially in comparison to CBCT. Whereas CBCT data of the specimen were reconstructed and analyzed with an isometric spatial resolution of 100 µm, the pixel size in mammography and tomosynthesis was 85 µm and therefore only slightly less than the resolution of CBCT. Although spatial resolution is generally lower at DBT, some studies indicate, that the detection and characterization of microcalcifications and associated carcinomas do not lead to significant differences between DBT and DM^18–22^. Previous studies showed reduced detectability of microcalcifications of dedicated breast CT systems in comparison to DM^27^, which could be improved over time with improvement of methods, so that most of even small microcalcifications are reproducible by CBCT systems^28,29^.

**Figure 4:**
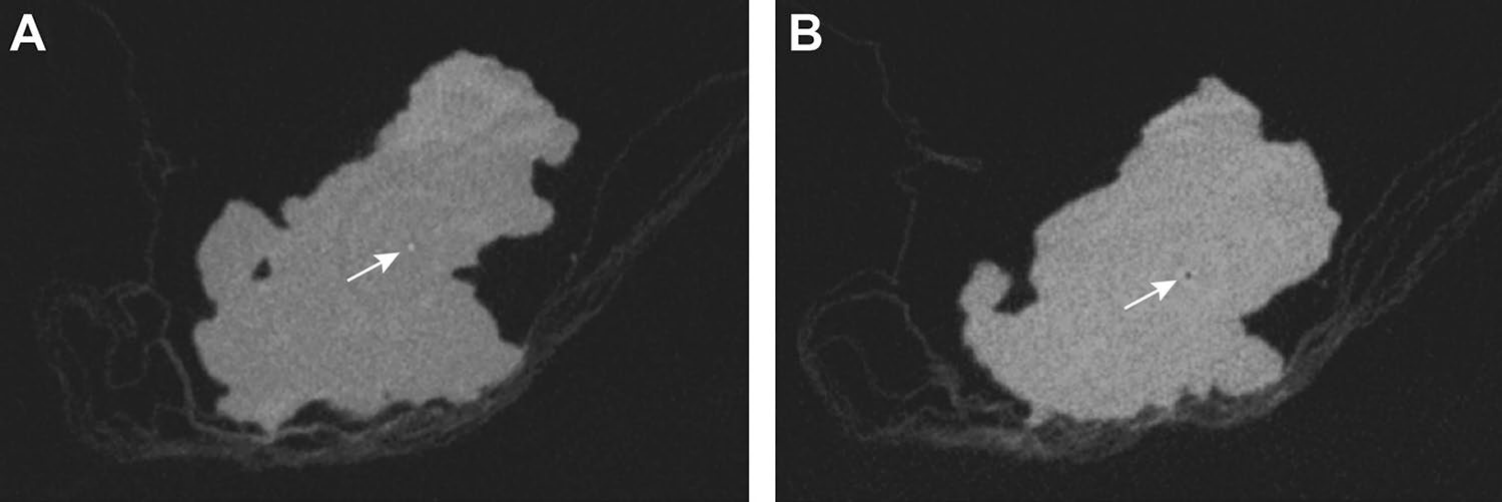
Artifacts in CBCT images of specimens were usually artifacts of the central axis that typically appeared as a central hyperdense spot in some slices (**A**) and a central hypodense spot in other slices (**B**) or ring artifacts (**A** and **B**).

The possible appearance of several artifacts in DBT like blurring-ripple artifacts with shadowing and zipper artifacts or bright-edge artifacts^12^ is known. In CBCT imaging beam hardening artifacts, exponential edge-gradient effects, aliasing or ring artifacts can appear^30^. With both methods some artifacts might reduce the visibility of microcalcifications, whilst others might even imitate the appearance of microcalcifications. We also noted artifacts with our CBCT system. In addition to ring artifacts (Fig. 4) or metal-related artifacts around the marking wire (Figs. 2C,D and 3B,C), they especially appeared in the middle of specimens. These central axis artifacts in CBCT images were represented as central hyperdense spots in some slices, that might have been misinterpreted as microcalcifications. But as they were always accompanied by a central hypodense spot in adjacent slices, this typical representation and the central location made it easy to be identified as an artifact (Fig. 4). Metal-related artifacts, for example due to beam hardening, were seen around the wires in CBCT and less in tomosynthesis and made it difficult to evaluate the adjacent tissue (Figs. 2C,D and 3B,C). This might impair the evaluation of directly adjacent microcalcifications. But as none of the mentioned artifacts appeared in DM, which is performed in two projections after wire marking, a location of microcalcifications close to wires can be preoperatively identified. This information might be taken into account, when assessing the resection margin of a specimen with CBCT and in case of unchanged wire location the according distance might be evaluated.

In addition, the visualization of microcalcifications in CBCT can be affected by scan parameters such as applied dose, tube voltage, detector quality or size of investigated specimens. Whereas the detectability of microcalcifications is significantly increased following a higher dose, it is decreased with more volume of the investigated object^31^. Therefore, the visibility of microcalcifications might be improved with carefully adapted scan protocols, reduced artifacts, improved detector and CT technology and better post imaging processing and reconstructions including metal artifact reduction.

Rößler et al. demonstrated that another advanced dedicated breast CT with photon-counting technology even showed a comparable or superior performance for the detection of microcalcifications and lesions in specimens compared to DM and DBT^32^.

Only recently, Wetzl et al.^33^ confirmed that the sensitivity and specificity for the detection of microcalcifications in breast specimens is at least equal or better for photon-counting breast CT compared to DBT with DM as standard reference. Therefore, with a dedicated breast CT system the visibility of microcalcifications in specimens might be further improved.

In addition, we found that CBCT was the most accurate method with the least measurement errors for determining the distance of microcalcifications, if present, to the resection margin with significant better results compared to DM and DBT. In our clinical practice a specimen is investigated with DM in two projections and the minimal detectable resection margin is measured on those images. In our study we equally investigated the specimens with DM in two projections. Still, the specimen was regularly simply placed only in the two main orientations (craniocaudal and mediolateral) directly on the investigating table. Accordingly, the distribution of the frequently irregular specimen on the investigating table follows the principles of gravitas with the possibility of unwanted tilts. Therefore, the specimen is object of not always influenceable and not anatomical correct superimpositions which altogether influence the result of the measurements in DM imaging. For DBT the specimen was equally placed on the investigation table, i.e. DBT was performed directly after the first DM projection with the same device and without repositioning of the specimen with 25 projections over a 50° angle. In contrast, in CBCT the specimen was carefully placed in a custom made and adaptive storage aid for investigation (compare Supplementary Fig. S1). Therefore, only with CBCT true volumetric positioning of the specimen could be performed. In addition, CBCT allows high contrast imaging with no tissue overlap and multiplanar reconstructions. This might explain the better distance measurements with CBCT than with DM and even with DBT in comparison to histopathological measurements as gold standard. It must however be conceded that histopathology itself is susceptible to a certain degree of error regarding the detection and analysis of microcalcifications in specimens. Microcalcifications might not be detected due to their small size or their location. However, in the case that microcalcifications known by mammography are not detected histopathologically, further radiographic examinations of the histology sections and the specimen and appropriate histopathological analyses were performed. Still, this might potentially be a source of error and further analysis in studies with more samples should be conducted.

The exposure time for all three methods varied in the range of seconds with 0.3 s for DM and up to 10 s for CBCT. For the investigation of specimens regarding microcalcifications and the measurement of the resection margin to microcalcifications there was no significant difference in reading time between DM and DBT in our study. Only CBCT imaging resulted in a significant longer reading time in comparison to DM or DBT. This is most likely owed to the greater amount of imaging data in CBCT due to its 3D acquisition and presentation of the specimens. Nevertheless, the difference is in the range of seconds and therefore does not relevantly delay the operation time until the result can be communicated to the breast surgeon. Therefore, the difference in examination and reading time might be tolerable considering the benefits of a more exact resection margin assessment and radiological report for the surgeon following CBCT.

Still, a breast CT system or another suitable CBCT as in our case has to be available and it should be located within easy reach of the operating theatre and the pathology department for immediate imaging during surgery, as the processing and transport of the specimen should not be significantly delayed due to a change in imaging modality. For even faster direct intraoperative imaging a dedicated specimen CT might be a conceivable future development. In contrast to other studies that proved dedicated breast CBCT dose to be similar to or even less than DM dose^24^, in our study, dose measurements showed a much higher dose for CBCT than for DM in two projections or DBT. As we only investigated specimens and as our CT system was not used for in vivo breast imaging this has no further relevance.

Regarding the latest promising studies with further improved breast CT imaging technology for specimens^32,33^ as well as for breast imaging, which might even be additionally improved by contrast media application^25^, more focus should be laid on breast CT. Especially in preoperative imaging dedicated breast CT might be a promising alternative to the more expensive breast MRI. In imaging of specimens, CBCT might soon become a realistic alternative for DM, especially as a cancer free margin of specimens is extremely important for later outcome of patients^10^. One step further, in combination with preoperative breast CT even more advantages might be expected by specimen CBCT. One may even speculate that the assessment of non-calcified tumor parts could possibly be easier in the postoperative CBCT examinations of the specimens, if they are compared with preoperative contrast-enhanced breast CT examinations. It can be assumed that this would improve the ability to assess resection margins in postoperative CBCT and help with the guidance of surgical decisions.

In addition, several other methods like micro-CT, specimen MRI, high frequency ultrasound, even PET or electromagnetic imaging and optical depiction are theoretically available for specimen analysis regarding the cancer detection and correct depiction of resection margins^34^. Certainly, a volumetric imaging with high contrast and spatial resolution and better reconstruction modes would be of advantage, but there is still room for quality improvement and more studies should underline these alternative ways.

This study is limited by its monocentric design. Due to the exclusion of some patients for several reasons, a selection bias might have occurred, although in our view this should not have had a relevant influence on the results of this study.

In conclusion, in this study we were able to show that measurement of the resection margin to microcalcifications was significantly more accurate with CBCT compared to DM and DBT, most likely due to better 3D-placement and analysis of specimens. A combination of methods or improving CT technology might be more promising for improved determination of disease-free margins of breast specimens with microcalcifications than DM alone. However, further studies have to be performed, especially regarding additional criteria for margin assessment of specimens like soft tissue changes associated with breast cancer.

## Methods

### Study population

In our study we enrolled consecutive patients who received breast surgery (ablation as well as breast conserving surgery) in our institution and who presented preoperatively to the Radiology Department over a period of six months. Subjects were excluded from participation if they were minor, declined participation, required frozen sectioning, were enrolled in different studies, had incomplete preoperative imaging or in case of interference with clinical workflow.

The Ethics Committee of the University of Freiburg approved this prospective study (No. 507/12), which was in accordance with the Helsinki Declaration and all subjects provided written informed consent.

### Radiologic examination of specimens

After breast surgery, resected specimens were immediately and without any further procession examined with DM, digital breast tomosynthesis, and cone-beam computed tomography. DM was performed with 24 kV and 9 mAs (Mammomat Inspiration, Siemens Healthineers, Erlangen, Germany) and included two projections after positioning the specimen with the help of suture markings in craniocaudal and mediolateral orientation. DBT was performed with 25 projections over a scan angle of 50 degrees with 24 kV and 71mAs in the same device directly after DM in craniocaudal orientation, therefore no repositioning of the specimen was necessary for DBT examination (Mammomat Inspiration, Siemens Healthineers, Erlangen, Germany). CBCT was performed with 96 kV and 36 mAs (Verity, Planmed Oy, Helsinki, Finland, originally designed for musculoskeletal imaging). Our CBCT scanner features an un-binned pixel-size of 127 µm at the detector with a magnification of 1.5, resulting in a geometrical resolution of 85 µm. Un-binned CBCT data of the specimen were acquired and reconstructed on an 100 µm isometric grid due to the given reconstruction algorithm. The pixel size of DM and DBT was 85 µm.

The exposure time for mammography was 0.3 s, for tomosynthesis 2.4 s and for CBCT 10 s.

Dose measurement was performed for DM, DBT and CBCT using thermoluminescent dosimeter (TLD), that were centrally placed in an imaging phantom of breast specimen like amorphous tissue consisting of 100 g Gallus gallus domesticus fatty and muscular tissue. Subsequently TLDs were analyzed with a TLD reader and the absorbed dose was calculated.

Positioning in the CBCT was done with a custom made styrofoam storage aid. Oriented to the suture markings, this allowed an axially correct examination of the specimens.

Directly after the radiologic investigation the specimens were stored in formaldehyde and analyzed at the pathological institute.

After screening the first 50 patients we performed an interim analysis. In this analysis the primary endpoint of the study was met, and the trial was closed.

### Radiologic analysis of preoperative mammography and specimens

The result of the histopathological examination served as the reference standard for resection margin. A consensus reading of mammography by two radiologists with over 25 and 5 years of experience in breast imaging served as the reference standard for the presence of microcalcifications.

The images of the examinations were pseudonymized and submitted to three blinded raters in a randomized fashion. All raters were breast radiologists with an experience in breast imaging of 7, 8 and 26 years. The raters assessed the examinations of the different imaging modalities at least 4 weeks apart to avoid recall bias. In each evaluation cycle, the studies were randomized in a different order. Image evaluation was performed on approved diagnostic monitors under constant light conditions. The assessment criteria included the presence of microcalcifications and their diagnostic reliably (judged by Likert scale 1–4 with 1 being very safe and 4 being very uncertain). Only if microcalcifications could be delineated, the smallest distance of those microcalcifications to the resection margin was measured (in mm, compare Fig. 2) and the accessibility of this measurement was evaluated (Likert scale 1–4 with 1 being very good accessibility and 4 being very poor accessibility). The reading time was recorded using a stopwatch.

In addition, one radiologist measured the maximal size of microcalcifications in the mammography of all specimens.

### Standard pathological assessment

Gross section was conducted according to standardized routine gross section protocols. After formalin fixation (minimum 24 h) all tissue specimens were macroscopically examined and documented. Resection margins (medial, lateral, cranial, caudal, ventral and dorsal) were ink-dyed. Next, sections of 0.4 cm thickness were cut and observed for macroscopic conspicuities by an experienced pathologist. Tissue specimens with a maximal diameter of 3.0 cm or less were completely embedded. From tissue specimens with a diameter above 3.0 cm macroscopic conspicuous areas as well as clinical labeled areas were embedded with reference to the nearest resection margins. Of note, macroscopic conspicuous areas were individually described, measured in three dimensions and documented in relation to all nearest resection margins.

After formalin fixation and paraffin embedding, tissue slices of 3 μm thickness were automatically Hematoxilin and Eosin stained using the Dako Cover Stainer.

All slides were histologically examined by an experienced pathologists for histological abnormalities. Histopathological reports included histological findings (e.g. usual ductal hyperplasia, fibrocystic change and columnar cell changes). Microcalcifications, defined as deposits below 0.5 mm, were identified using HE staining and documented as far as histologically detectable. If, despite clinical "indication of microcalcification", no microcalcifications were histologically detectable, supplementary sectional stages were prepared. If the result was not sufficient, an X-ray of all blocks was requested and corresponding blocks were completely serially sectioned and HE stained.

In case of a malignant tumor, histological reporting comprised WHO classification, UICC stage, tumor grading according to Silverstein (DCIS) and Elston & Ellis (tumor grading), the presence or absence of lymphatic, vascular and perineural invasion and the histological assessment of the medial, lateral, cranial, caudal, ventral and dorsal resection margin in cm using an ocular micrometer.

In the case of a differential diagnosis of UDH/DCIS/invasive carcinoma, additional immunohistochemical tests were performed for Cytokeratin 5/6, p63, oestrogen receptor protein, progesterone receptor protein, HER2/neu and MIB1.

### Statistical analysis

Statistical analysis and presentation of the collected data were performed using the statistical program R version 4.0.2 (The R Foundation for Statistical Computing).

Descriptive data were presented using absolute frequency, mean value (MV) and confidence intervals (CI).

Inter-rater reliability for the detection of microcalcifications were calculated with Fleiss kappa. Inter-rater reliability for the measurements of resection margin were calculated with intraclass correlation (twoway, agreement). The error in the measurement of the resection margin was calculated by subtracting the evaluators' measurements from the reference standard. The error of measurement, the reading time, the confidence for detection of microcalcifications and the confidence for measurement of microcalcifications were compared between the three modalities with Friedman rank sum test and post hoc pairwise comparison using Nemenyi multiple comparison test.

Sensitivity and specificity were calculated for the detection of microcalcifications for each modality separately pooled for all raters. P-values were corrected with Bonferroni adjustment. A *p*-value < 0.05 was considered significant.

## Supplementary Information

Supplementary Information.

## Data Availability

The datasets generated and analyzed during the current study are available from the corresponding author on reasonable request.
