# Supplementary material for: Accuracy of cone-beam computed tomography, digital mammography and digital breast tomosynthesis for microcalcifications and margins to microcalcifications in breast specimens
Source: Sci Rep. 2022 Oct 21;12:17639. doi: 10.1038/s41598-022-21616-3 (PMC9587219; doi:10.1038/s41598-022-21616-3)
Supplement: Supplementary file 1 — Supplementary Information. [file 41598_2022_21616_MOESM1_ESM.docx]

Supplementary Figure S1


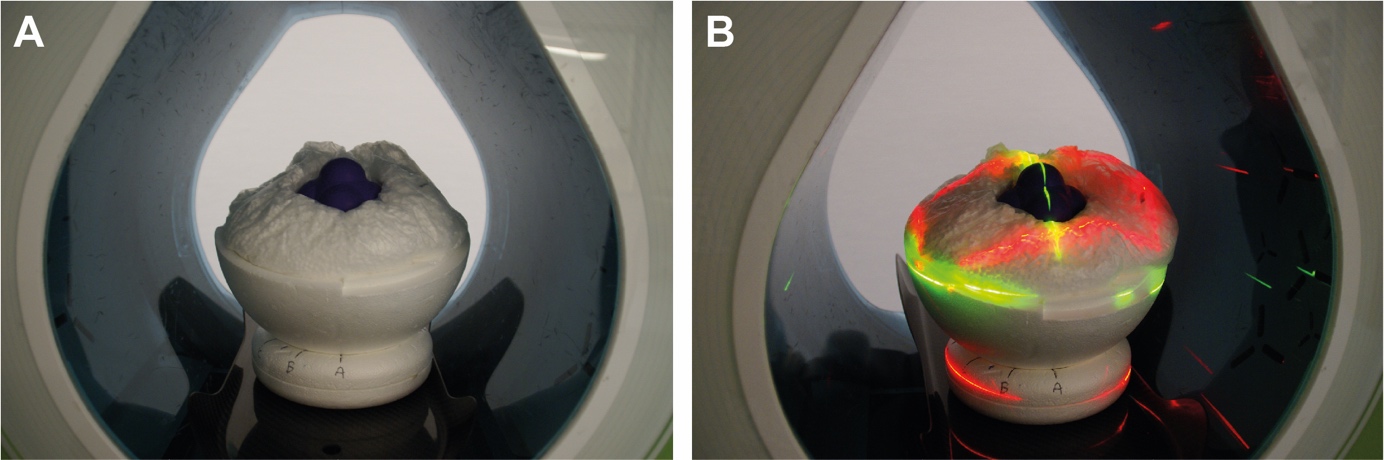


Figure S1: Positioning of specimens (a dummy was used for these pictures) in the gantry of the cone-beam computed tomography (CBCT) in our custom made and adaptive storage aid for investigation made of styrofoam spheres in a styrofoam container (A and B). In this way true volumetric positioning of the specimen could be performed.
